# Roots of resilience: positive childhood experiences, sense of belonging, and burnout among medical students

**DOI:** 10.3389/fpubh.2026.1857198

**Published:** 2026-06-25

**Authors:** Tina Izad, Lauren Walkon, Rachel Lloyd, Michael Nazmifar, Rebecca Bienstock, Changiz Mohiyeddini

**Affiliations:** Department of Foundational Medical Studies, Oakland University William Beaumont School of Medicine, Rochester, MI, United States

**Keywords:** burnout, mediation, medical students, positive childhood experiences, sense of belonging, year of training

## Abstract

**Background:**

Burnout affects many medical students, yet the role of positive childhood experiences (PCEs) remains largely unexplored. Sense of belonging protects against burnout, but whether it mediates the link between PCEs and burnout is unknown.

**Objective:**

To test whether sense of belonging mediates the association between PCEs and burnout in medical students.

**Method:**

This cross-sectional survey study examined medical students (N = 132) at a U. S. medical school using validated measures of PCEs, sense of belonging, and burnout. Mediation was tested using PROCESS Model 4 with 5,000 bias-corrected bootstrap confidence intervals. Year of training, age, and gender were covariates.

**Results:**

All primary bivariate correlations were significant (*p* < 0.001). PCEs predicted belonging (B = 1.389, *β* = 0.353, *p* < 0.001), and belonging predicted lower burnout controlling for PCEs and covariates (B = −0.490, *β* = −0.555, *p* < 0.001). The indirect effect was significant (B = −0.680, *β* = −0.205, 95% BootCI [−1.124, −0.297]). The direct effect remained significant (B = −0.806, *p* = 0.001), indicating partial mediation. The model explained 51.1% of burnout variance; belonging accounted for 45.8% of the total PCE − burnout association. Age was significant (*p* = 0.026); year of training showed a marginal trend toward higher burnout in more advanced students (*p* = 0.065).

**Conclusion:**

Findings are consistent with a partial mediation model in which belonging explains a substantial portion of the PCE − burnout association. Since belonging is institutionally modifiable, fostering belonging-oriented environments offers an evidence-informed burnout prevention strategy, especially for students with fewer nurturing childhood experiences.

## Introduction

Medical education is widely recognized as one of the most demanding academic and professional training environments in modern society. The combination of cognitive overload, high-stakes assessment, early clinical exposure to suffering and death, and the sustained suppression of personal needs creates a context uniquely conducive to psychological exhaustion. Burnout — defined by the World Health Organization’s ICD-11 as a syndrome arising from chronic workplace stress characterized by energy depletion or exhaustion, increased mental distance from one’s professional role, and reduced professional efficacy — has emerged as a critical public health concern within medical education globally (1). Systematic review evidence indicates that burnout affects between 40 and 80% of medical students, with rates consistently exceeding those observed in age-matched peers outside medicine (2, 3). In the United States alone, approximately half of all medical students report burnout symptoms at any given time (4). The magnitude of this problem demands a theoretically grounded research agenda that moves beyond documenting prevalence to identifying the developmental and contextual mechanisms that render certain students more vulnerable — or more resilient — in the face of the rigors of medical training.

Research on childhood antecedents of adult psychological health has long been dominated by the adverse childhood experiences (ACEs) framework (5). A growing body of scholarship now centers positive childhood experiences (PCEs) — defined as experiences of safe, stable, and nurturing relationships and environments during childhood (6) — as independent protective resources. Using a large population-representative sample, Bethell et al. (7) demonstrated that higher PCE scores predicted significantly better adult mental and relational health, including reduced rates of depression and anxiety, even among individuals with high ACE exposure and after controlling for current social support. The systematic review by Narayan et al. (8), synthesizing 58 empirical studies, confirmed that PCEs are associated with reduced depressive, anxiety, and post-traumatic stress symptoms, with 24 studies finding these effects held after controlling for childhood adversity. Despite this growing literature, the application of the PCE framework to the specific population of medical trainees remains strikingly limited.

Sense of belonging — broadly defined as the subjective experience of feeling accepted, valued, and included within one’s social and institutional environment — has long been theorized as a fundamental human psychological need (9). In medical education specifically, Leep Hunderfund et al. (10), in a landmark cross-sectional study published in Academic Medicine, found that learners with very strong organizational belonging demonstrated substantially lower odds of burnout (OR = 0.05; 95% CI: 0.02–0.12) compared to those reporting weak belonging. A parallel study among Chinese medical residents (11) similarly found that higher sense of school belonging served as a significant protective factor against emotional exhaustion. In qualitative research with early-year medical students, Prendergast et al. (12) found that peer-support groups and group problem-based learning generated a sense of community that meaningfully reduced burnout — consistent with the view that belonging operates as an active protective factor against the chronic stressors of medical training.

If sense of belonging is a potent protective factor against burnout, a theoretically important question concerns where the capacity for belonging originates. Attachment theory offers one compelling answer: the early relational experiences that constitute PCEs — having at least one safe caregiver, predictable home routines, trusted friendships, and environments characterized by fairness and security — are precisely the experiences through which children develop internal working models of relationships as trustworthy, reciprocal, and nurturing (13, 14). From a developmental psychopathology perspective, PCEs scaffold the development of social competence, interpersonal trust, and emotional regulation — each of which is a prerequisite for experiencing authentic belonging in demanding, hierarchically structured environments such as medical schools (8, 15). Recent research by Willis et al. (16) found that young adults with higher PCEs reported stronger social connectedness controlling for ACEs, providing empirical support for the hypothesis that PCEs shape belonging as a developmental outcome, which in turn cascades into professional well-being.

To understand why medical students are disproportionately affected by burnout, it is necessary to situate their experience within Maslach and Leiter's (17) Job Demands-Resources (JD-R) model, which posits that burnout arises from a chronic imbalance between job demands and job resources. Medical students face an extreme version of this imbalance, with vast curricular demands and insufficient institutional support or peer connection (18). Among the most salient risk factors for burnout are perceived lack of social support, poor peer relations, and a low sense of belonging (10, 18). Sense of belonging was selected as the mediator in the present study over other plausible candidates — such as emotion regulation or self-compassion — because it is both theoretically proximal to the developmental pathway from PCEs (early relational experiences specifically scaffold institutional integration) and practically modifiable at the institutional level, making it the mechanism with the most direct implications for evidence-based burnout prevention.

Against this backdrop, the present investigation proposes an integrated developmental-contextual model in which PCEs exert both direct and indirect effects on burnout, with sense of belonging operating as a mediating psychological pathway. This model integrates attachment theory (13), developmental psychopathology (8, 15), and the JD-R model (17). While PCEs are retrospective and not directly amenable to intervention, sense of belonging is a potentially modifiable institutional resource. We acknowledge at the outset that the present model does not control for all potential confounders, including socioeconomic background, prior mental health history, and neuroticism; the implications of this are addressed in the Limitations section. The present study tested three hypotheses using cross-sectional data. All hypotheses are stated in terms of statistical associations; mediation here refers to statistical mediation — a pattern of associations consistent with an indirect pathway — rather than confirmed temporal or causal mediation.

*H1*: PCEs will be negatively associated with burnout.

*H2*: PCEs will be positively associated with sense of belonging.

*H3*: Sense of belonging will statistically mediate the cross-sectional association between PCEs and burnout, such that part of the association between PCEs and lower burnout will be explained by the positive association between PCEs and belonging and the negative association between belonging and burnout.

## Method

### Participants and procedure

A cross-sectional survey design was employed to examine the relationships among PCEs, sense of belonging, and burnout among medical students. A census recruitment approach was used: survey invitations were distributed via institutional email to all enrolled students across all four years of the Oakland University William Beaumont School of Medicine (OUWB) MD program, comprising approximately 640 enrolled students. Follow-up reminder emails were sent to maximize response. Of the 171 students who initiated the survey, 39 were excluded due to missing data on one or more study variables (including year of training), yielding a final analytic sample of N = 132 following consistent listwise deletion across all analyses (approximate response rate: 21%). All analyses used the same 132-participant sample to ensure consistency across descriptive statistics, correlations, and the mediation model.

The final analytic sample comprised medical students with a mean age of 25.26 years (SD = 1.98, range = 21–36). Gender distribution was 61.4% female, 37.9% male, and 0.8% non-binary or third gender. Year of enrollment spanned all four years of the MD curriculum: M1 = 14 (10.6%), M2 = 63 (47.7%), M3 = 40 (30.3%), and M4 = 15 (11.4%). The majority of the sample (58.3%) were in preclinical training years (M1 and M2). All study procedures were reviewed and approved by the institutional review board (IRB-FY2025-287). Participation was entirely voluntary and anonymous; no compensation was provided. Inclusion criteria: current enrollment in the OUWB MD program. Exclusion criteria: failure to complete any study variable including covariates.

### Measures

#### Positive childhood experiences

Positive childhood experiences were assessed using the Benevolent Childhood Experiences (BCEs) Scale (19), a 10-item binary measure in which participants indicated Yes or No to questions about protective and nurturing experiences during their first 18 years of life (e.g., ‘Did you have at least one caregiver with whom you felt safe?’; ‘Did you have a predictable home routine, like regular meals and a regular bedtime?’). The total score was computed as the proportion of items endorsed, yielding a continuous scale ranging from 0 to 1.0, with higher scores reflecting a greater accumulation of positive childhood experiences. The BCEs has demonstrated good predictive validity across multiple adult samples, with higher scores associated with reduced depression, anxiety, and post-traumatic stress symptoms independent of childhood adversity levels (8, 20). In the present sample, M = 0.83, SD = 0.25, alpha = 0.86.

#### Sense of belonging

Sense of belonging was assessed using the Sense of Belonging Scale (21), a 5-item measure assessing students’ subjective experience of comfort, acceptance, support, commitment, and inclusion within their program environment (e.g., ‘I feel I am a part of this program’; ‘I am accepted at this program’). Items were rated on a 5-point Likert scale ranging from 1 (strongly disagree) to 5 (strongly agree), with higher scores reflecting stronger sense of belonging. The scale has demonstrated good reliability and validity across diverse educational settings (21, 22). In the present sample, M = 3.20, SD = 1.00, alpha = 0.87.

#### Burnout

Burnout was assessed using the Maslach Burnout Inventory-Student Survey [MBI-SS; (23)], a 15-item measure capturing three dimensions of student burnout: Emotional Exhaustion (five items; e.g., ‘I feel emotionally drained by my studies’), Cynicism (four items; e.g., ‘I doubt the significance of my studies’), and Academic Efficacy (six items, reverse-scored; e.g., ‘I feel confident in my ability to successfully finish my degree’). Items were rated on a 7-point frequency scale ranging from 0 (never) to 6 (every day). Academic Efficacy items were reverse-scored prior to analysis such that higher values on all subscales reflected greater burnout severity. A composite burnout score was computed by averaging across the three subscale means, with higher scores indicating greater burnout severity. The use of a composite score is consistent with prior medical education burnout research treating global burnout severity as a theoretically meaningful unitary construct (2, 3, 18), supported by confirmatory factor analytic work demonstrating a higher-order burnout factor across MBI-SS subscales (23). In the present sample, M = 2.67, SD = 0.86, alpha = 0.91.

### Analytic strategy

Prior to primary analyses, standard assumptions for parametric regression were evaluated. Residual plots were inspected for linearity and homoscedasticity; no systematic violations were detected. All variance inflation factors (VIFs) were below 2.0, indicating acceptable multicollinearity. The BCEs variable exhibited a pronounced positive skew (M = 0.83, SD = 0.25). As a robustness check, Spearman rank-order correlations were computed alongside Pearson correlations; results were virtually identical (PCEs-Burnout: rho = −0.442 vs. r = −0.442; PCEs-Belonging: rho = −0.353 vs. r = 0.353; Belonging-Burnout: rho = −0.659 vs. r = −0.659), confirming that the skew did not materially distort the bivariate associations. All analyses used consistent listwise deletion across all study variables including covariates, ensuring that all results — descriptive statistics, bivariate correlations, and the mediation model — are based on the same analytic sample of *N* = 132.

Bivariate relationships among PCEs, sense of belonging, and burnout were examined using Pearson product–moment correlations (two-tailed). The mediation hypothesis was tested using Hayes' (24) PROCESS macro for SPSS (Model 4, simple mediation) with 5,000 bias-corrected bootstrap confidence intervals at a 95% confidence level (seed = 12,345). Year of training, age, and gender were entered as covariates in both equations of the mediation model. PCEs served as the independent variable (X), sense of belonging as the mediating variable (M), and burnout as the dependent variable (Y). An indirect effect is interpreted as statistically significant when its 95% bias-corrected bootstrap CI excludes zero (24, 25). All analyses were conducted in SPSS Statistics.

### *A priori* power analysis

An *a priori* power analysis was conducted prior to data collection. Because PROCESS does not offer a dedicated mediation power module, we followed the approach recommended by Fritz and MacKinnon (26) and used Monte Carlo simulation methods appropriate for indirect effect estimation (27). Based on the prior PCE literature (7, 8), we assumed a medium indirect effect (standardized beta approximately −0.15 to −0.20). Simulations indicated a minimum required sample of approximately *N* = 100–120 to achieve power = 0.80 at alpha = 0.05. The final analytic sample of *N* = 132 exceeds this threshold. Moreover, the observed path coefficients in the present sample (path a B = 1.389; path b B = −0.490) reflect medium-to-large effects, suggesting that the achieved sample provides adequate and likely conservative power for the primary mediation test.

### Missing data analysis

Of the 171 participants who initiated the survey, 39 were excluded due to missing data on one or more study variables including year of training, yielding a final analytic sample of *N* = 132. Little's (28) MCAR test confirmed that data were missing completely at random, chi-squared (2) = 0.099, *p* = 0.952, indicating that listwise deletion is an unbiased analytic strategy. EM-estimated correlations were virtually identical to listwise-derived correlations, providing further confirmation that the pattern of missing data did not meaningfully distort the observed associations.

## Results

### Descriptive statistics and bivariate correlations

Table 1 displays the sociodemographic make up of the sample.

**Table 1:** Demographic characteristics of the sample (*N* = 132).

Characteristic	*n*	%	Valid %
Age (years): M = 25.26, SD = 1.98, Range = 21–36			
Gender			
Female	81	61.4%	61.4%
Male	50	37.9%	37.9%
Non-binary/third gender	1	0.8%	0.8%
Year of Training			
M1 (First year)	14	10.6%	10.6%
M2 (Second year)	63	47.7%	47.7%
M3 (Third year)	40	30.3%	30.3%
M4 (Fourth year)	15	11.4%	11.4%
Sample			
Initial respondents	171	100%	—
Excluded (missing data on primary measures)	26	15.2%	—
Excluded (missing year of training)	13	7.6%	—
Final analytic sample (listwise)	132	77.2%	—

All demographic statistics are based on the final analytic sample of *N* = 132 following consistent listwise deletion across all study variables. Gender and year distributions reflect valid responses within this sample.

Bivariate correlations among the study variables are presented in Table 2. All analyses are based on the consistent listwise analytic sample of *N* = 132. Medical students reported a mean PCE score of M = 0.83 (SD = 0.25), indicating that on average participants reported approximately 83% of assessed positive childhood conditions. Mean sense of belonging was M = 3.20 (SD = 1.00), reflecting moderate-to-strong institutional belonging. Mean burnout was M = 2.67 (SD = 0.86), consistent with moderate burnout levels documented in recent medical student samples (2, 18).

**Table 2:** Descriptive statistics and intercorrelations among study variables (*N* = 132).

Variable	Min	Max	M	SD	1	2	3
1. Positive childhood experiences	0.00	1.00	0.83	0.25	—		
2. Sense of belonging	0.72	4.72	3.20	1.00	0.353**	—	
3. Burnout	1.00	4.00	2.67	0.86	−0.442**	−0.659**	—

PCEs = Positive childhood experiences (mean score 0–1). Belonging range = 0.72–4.72; Burnout range = 1.00–4.00. Correlations are Pearson r based on the consistent listwise analytic sample (*N* = 132). **p < 0.01 (two-tailed).

All three primary bivariate correlations were statistically significant at *p* < 0.001. PCEs were negatively and moderately associated with burnout (r = −0.442, *p* < 0.001). PCEs were positively associated with sense of belonging (r = +0.353, *p* < 0.001). Sense of belonging demonstrated the strongest association with burnout (r = −0.659, *p* < 0.001), consistent with findings from Leep Hunderfund et al. (10). Year of training was significantly associated with burnout (r = 0.220, *p* = 0.011) and with belonging (r = −0.179, *p* = 0.040), indicating that more advanced students reported higher burnout and lower belonging. The pattern and magnitude of these associations provided strong preliminary support for the hypothesized mediation model.

### Mediation analysis: sense of belonging as a mediator

**Figure 1:**
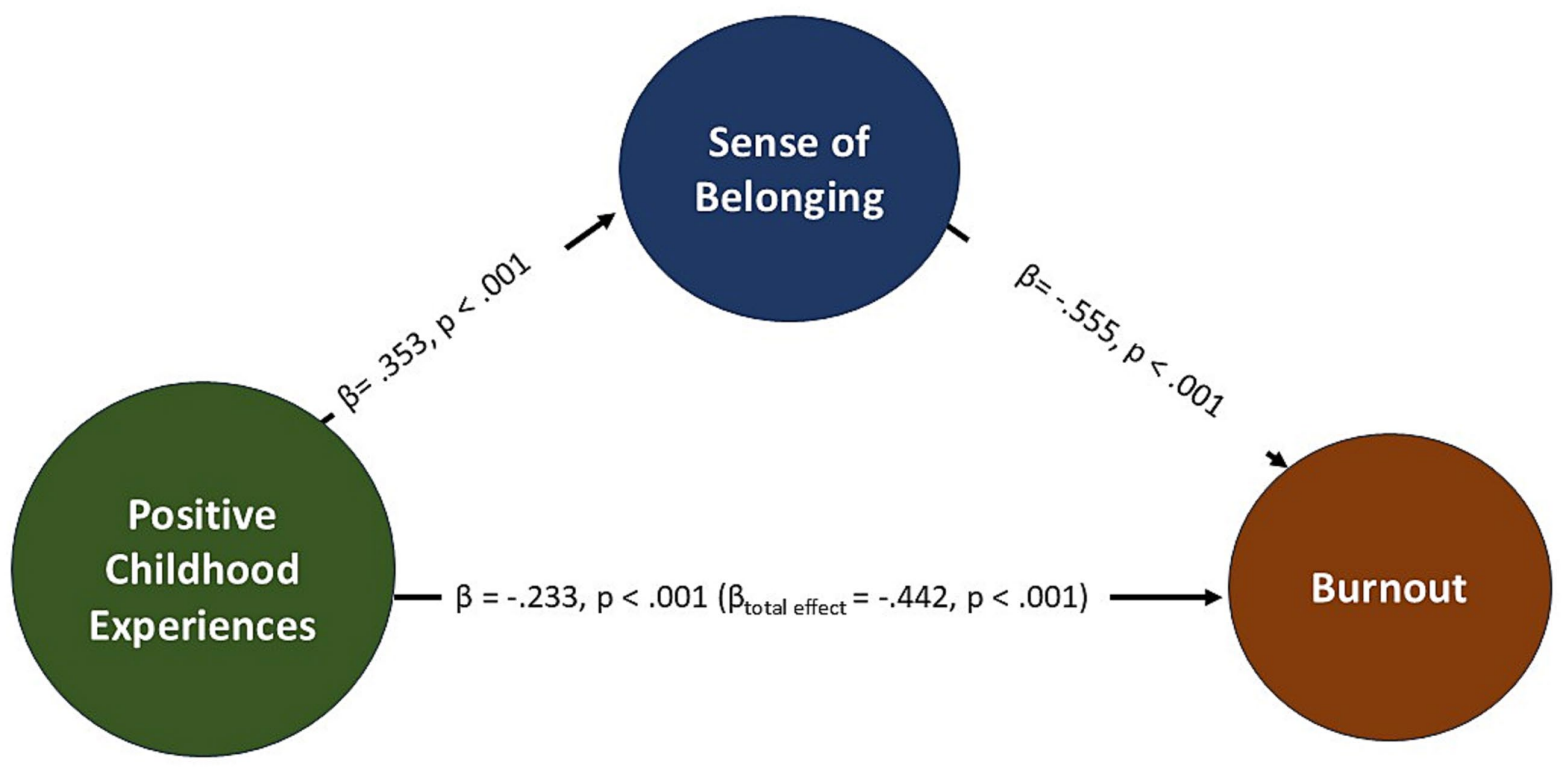
Standardized path coefficients for the mediation model. Sense of belonging significantly mediated the relationship between positive childhood experiences and burnout in medical students (N = 132). Values on paths are standardized regression coefficients (*β*). The total effect of PCEs on burnout is shown in parentheses (β = −0.442, *p* < 0.001). Path a: β = 0.353, *p* < 0.001. Path b: β = −0.555, *p* < 0.001. Direct effect (c’): β = −0.233, *p* = 0.001. The indirect effect (a × b) was significant, β = −0.205, 95% bias-corrected bootstrapped CI [−1.124, −0.297], indicating partial mediation. Sense of belonging accounted for 45.8% of the total effect. Mediation model controlled for year of training, age, and gender. ***p < 0.001.

The mediation model was tested using PROCESS Model 4 (24) with 5,000 bias-corrected bootstrap confidence intervals, controlling for year of training, age, and gender (*N* = 132). Results are summarized in Table 3 and depicted in Figure 1.

**Table 3:** Regression coefficients for the mediation model with covariates (PROCESS model 4, *N* = 132).

Variable	B	SE	β	t	p	LLCI	ULCI
Outcome: sense of belonging R^2^ = 0.139, F (4, 127) = 5.11, *p* < 0.001							
Constant	2.853	1.283	—	2.223	0.028	0.313	5.392
PCEs (path a)	1.389	0.352	0.353	3.949	< 0.001	0.693	2.085
Year of training	−0.124	0.107	−0.117	−1.156	0.250	−0.335	0.088
Age	−0.016	0.048	−0.030	−0.335	0.738	−0.112	0.079
Gender	−0.055	0.164	−0.029	−0.333	0.740	−0.379	0.270
Outcome: burnout R^2^ = 0.511, F (5, 126) = 26.29, *p* < 0.001							
Constant	6.556	0.849	—	7.718	< 0.001	4.875	8.236
Sense of belonging (path b)	−0.490	0.058	−0.555	−8.496	< 0.001	−0.604	−0.376
PCEs (path c’)	−0.806	0.242	−0.233	−3.330	0.001	−1.285	−0.327
Year of training	0.130	0.070	0.122	1.865	0.065	−0.008	0.268
Age	−0.071	0.031	−0.155	−2.254	0.026	−0.133	−0.009
Gender	−0.107	0.107	−0.062	−1.003	0.318	−0.318	0.104
Effect decomposition							
Total effect of PCEs on burnout (path c)	−1.486	—	−0.442	—	—	—	—
Direct effect of PCEs on Burnout (path c’)	−0.806	0.242	−0.233	−3.330	0.001	−1.285	−0.327
Indirect effect via Belonging (path a x b)	−0.680	0.211^a^	−0.205	—	—	−1.124	−0.297

Proportion of total effect mediated: ab/c = −0.680/−1.486 = 45.8%. B = unstandardized regression coefficient; SE = standard error; beta = standardized coefficient; LLCI/ULCI = lower/upper limits of 95% confidence interval. For direct and total effects, CIs are based on OLS t-distribution. For the indirect effect (path a x b), LLCI/ULCI are bias-corrected bootstrap 95% CIs based on 5,000 resamples (seed = 12,345). CI excluding zero indicates significant indirect effect. an = 5,000 bias-corrected bootstrap replications. ^a^For the indirect effect, the standard error was estimated using 5,000 bias-corrected bootstrap resamples.

#### Path a — PCEs predicting sense of belonging

Positive childhood experiences were a significant positive predictor of sense of belonging (B = 1.389, beta = 0.353, SE = 0.352, t (127) = 3.949, *p* < 0.001, 95% CI [0.693, 2.085]). The model accounted for 13.9% of the variance in sense of belonging (R^2^ = 0.139, *F* (4, 127) = 5.11, *p* < 0.001). Year of training (B = −0.124, *p* = 0.250), age (B = −0.016, *p* = 0.738), and gender (B = −0.055, *p* = 0.740) were not significantly associated with belonging, indicating that the PCE-belonging association was not confounded by these demographic variables.

#### Path b — sense of belonging predicting burnout, controlling for PCEs

Sense of belonging was a significant negative predictor of burnout after controlling for PCEs and all covariates (B = −0.490, beta = −0.555, SE = 0.058, t (126) = −8.496, *p* < 0.001, 95% CI [−0.604, −0.376]). Greater belonging was associated with substantially lower burnout, consistent with the JD-R model framework in which social integration functions as a job resource protecting against exhaustion and depersonalization.

#### Path c’ — direct effect of PCEs on burnout, controlling for sense of belonging

PCEs retained a significant direct negative effect on burnout after sense of belonging and all covariates were entered (B = −0.806, beta = −0.233, SE = 0.242, t (126) = −3.330, *p* = 0.001, 95% CI [−1.285, −0.327]). Among the covariates, age was a significant predictor of burnout (B = −0.071, beta = −0.155, *p* = 0.026), with older students reporting slightly lower burnout. Year of training showed a marginal, non-significant trend toward higher burnout in more advanced students (B = 0.130, beta = 0.122, *p* = 0.065, 95% CI [−0.008, 0.268]). Gender was not significantly associated with burnout (B = −0.107, *p* = 0.318). The full model explained 51.1% of the variance in burnout (R^2^ = 0.511, *F* (5, 126) = 26.29, *p* < 0.001).

#### Indirect effect — mediation via sense of belonging

The indirect effect of PCEs on burnout through sense of belonging was B = −0.680 (beta = −0.205, Boot SE = 0.211, 95% bias-corrected BootCI [−1.124, −0.297]). Because the 95% confidence interval did not include zero, the indirect effect was statistically significant. The direct effect of PCEs on burnout remained significant (c’ = −0.806, *p* = 0.001), indicating partial rather than full mediation. Sense of belonging therefore represents one significant pathway through which PCEs are associated with burnout vulnerability in medical students, alongside other mechanisms not captured in the present model. The standardized indirect effect (beta = −0.205) falls in the medium range per conventional benchmarks (29). The standardized path coefficients for path a (beta = 0.353) and path b (beta = −0.555) both fall in the medium-to-large range. Belonging accounted for 45.8% of the total PCE-burnout association (indirect effect/total effect = −0.680/−1.486), representing a practically meaningful proportion given that belonging, unlike PCEs, is institutionally modifiable.

## Discussion

The present study examined the associations among positive childhood experiences, sense of belonging, and burnout in a sample of medical students, testing the hypothesis that sense of belonging statistically mediates the cross-sectional association between PCEs and burnout. The results provided robust support for this model. PCEs were significantly and negatively associated with burnout, significantly and positively associated with sense of belonging, and sense of belonging was in turn significantly and negatively associated with burnout — constituting the prerequisite pattern for mediation. The indirect effect of PCEs on burnout via belonging was statistically significant (B = −0.680, beta = −0.205, 95% bias-corrected BootCI [−1.124, −0.297]) and robust to control for year of training, age, and gender. The direct effect of PCEs also remained significant (B = −0.806, *p* = 0.001), confirming partial mediation. Taken together, these findings are consistent with a theoretical model in which early-life developmental experiences are associated with professional psychological well-being in medical trainees, with sense of belonging emerging as a statistically significant and practically meaningful indirect pathway.

The finding that PCEs are significantly associated with lower burnout among medical students aligns with and extends the broader PCE literature. Bethell et al. (7) demonstrated that higher PCEs predicted better adult mental health — including lower rates of depression and anxiety — even in individuals with histories of childhood adversity. The systematic review by Narayan et al. (8) identified PCEs as protective against a broad range of adult psychopathology. The present study extends this literature into the specific context of professional training stress and burnout, a domain where PCE research has been virtually absent.

The significant positive association between PCEs and sense of belonging (r = +0.353, path a B = 1.389, *p* < 0.001) is theoretically coherent and consistent with attachment-developmental perspectives. Bowlby's (13) attachment theory and developmental psychopathology scholarship (8, 15) posit that early experiences of safety, responsiveness, and relational nurturing generate internal working models of relationships as trustworthy and predictable — templates that are theorized to carry forward into adulthood and may shape the ease with which individuals forge connections in new social and institutional contexts. Our data are consistent with the interpretation that students who report enriched childhood environments may arrive at medical school with relational resources that facilitate integration into the professional community, though the cross-sectional design cannot confirm this developmental pathway.

The large negative association between sense of belonging and burnout (r = −0.659, path b B = −0.490, beta = −0.555, *p* < 0.001) is the strongest bivariate association in the matrix and corroborates the landmark findings of Leep Hunderfund et al. (10), who documented that very strong organizational belonging was associated with dramatically reduced odds of burnout (OR = 0.05) in a large multi-site sample. The standardized path b coefficient (beta = −0.555) falls in the large range, underscoring belonging as a potent proximal predictor of burnout. The qualitative work by Prendergast et al. (12) similarly identified sense of community as a meaningful protective factor against burnout in early-year medical students, lending ecological validity to the quantitative finding here.

The direct effect of PCEs on burnout remained significant and substantial after accounting for belonging, indicating that sense of belonging is not the sole pathway through which childhood experiences are associated with burnout vulnerability. This partial mediation pattern is consistent with a multi-pathway theoretical framework in which PCEs shape multiple psychological resources simultaneously — including but not limited to belonging. Adaptive emotion regulation (30), greater psychological flexibility, and stronger internal coping resources are among the likely additional pathways. The partial mediation finding invites future research to construct more fully specified models employing parallel or serial multi-mediator approaches to disentangle the relative contributions of belonging, emotion regulation, and self-compassion to the PCE-burnout relationship.

An important finding in the present model concerns year of training. Year of training showed a marginal positive trend in relation to burnout (B = 0.130, *p* = 0.065), suggesting a pattern consistent with higher burnout in more advanced students that did not reach conventional significance in the present sample. This trend is consistent with a well-established pattern in the medical education literature: burnout is not static across the curriculum but tends to escalate as students progress from preclinical to clinical training years. Longitudinal data document significant burnout escalation across the first year of medical school (31), and students in clinical training years consistently report higher emotional exhaustion and depersonalization than their preclinical peers, reflecting the cumulative stress burden of increasing patient care responsibilities and confrontation with clinical uncertainty (3). In the present sample, 41.7% of students were in clinical training years (M3 and M4). The marginal year effect, while not statistically significant at alpha = 0.05, is directionally consistent with this literature and warrants investigation in larger samples. Notably, year of training was not significantly associated with sense of belonging in the belonging equation (B = −0.124, *p* = 0.250), suggesting that the PCE-belonging pathway may be relatively stable across training phases. The bivariate correlation between year and belonging was significant (r = −0.179, *p* = 0.040), however, indicating that more advanced students do report somewhat lower belonging overall — a finding that has important practical implications for belonging-oriented interventions that target the critical preclinical-to-clinical transition.

Viewed in practical terms, the mediation finding carries an important implication: while positive childhood experiences are historically determined and not directly amenable to clinical or institutional intervention, sense of belonging is. The finding that belonging explains 45.8% of the total PCE-burnout association suggests that institutional efforts to cultivate belonging may substantially replicate, at the professional training level, the developmental advantages that nurturing childhood environments confer. Peer mentorship programs, inclusive small-group pedagogies, longitudinal cohort models, faculty accessibility, and the systematic cultivation of psychologically safe learning climates are all institutional levers that may elevate belonging — particularly for students whose childhood environments were less enriching. This has equity implications: students from disadvantaged or adversity-marked backgrounds may be especially reliant on institutional belonging as a compensatory resource, making belonging-oriented curricular and cultural investments a matter of both wellness and equity. It should be noted that all variables were collected via self-report in a single survey session, raising the possibility that shared method variance inflated the observed associations; the true associations may therefore be somewhat smaller than those reported here.

### Limitations

Several limitations of the present study merit consideration. First, the cross-sectional design precludes causal inference. Although the mediation model is theoretically motivated and statistically significant, the direction of effects cannot be established from correlational data. It is possible that lower burnout facilitates greater sense of belonging, or that unmeasured third variables account for the observed associations. Longitudinal designs are required to establish temporal precedence.

Second, the sample was drawn from a single allopathic medical school in the United States, limiting generalizability. Medical education contexts vary considerably across institutional type, geographic region, national system, and cultural context (2). Cultural norms surrounding emotional expression, help-seeking, and social connectedness may shape both the experience of belonging and the development of psychological resilience. Future research should replicate this model in multi-site samples spanning different types of medical schools and national contexts.

Third, all measures were collected via self-administered questionnaire, raising the possibility of shared method variance inflating observed associations, social desirability effects (particularly for burnout), and retrospective recall bias affecting PCE reports. The retrospective nature of PCE measurement is a recognized limitation of the field (8). Future research might supplement self-report with informant data, physiological indicators, or ecological momentary assessment.

Fourth, while the achieved sample size of *N* = 132 provided adequate power for the tested model, it may be insufficient to detect smaller effects or to estimate more complex multi-mediator models. The listwise deletion of 39 cases from the original pool of 171 respondents reduces statistical power and may introduce selection bias if excluded participants differ systematically from retained cases, though the MCAR test provided reassurance in this regard.

Fifth, the current model does not include potential confounding variables such as prior mental health history, neuroticism, family socioeconomic status, academic pressure, or minority stress — each of which may account for variance in both PCEs and burnout outcomes. Future research should incorporate comprehensive assessments of these variables to produce more conservative and causally credible effect estimates.

Sixth, the distribution of PCE scores exhibited a pronounced positive skew (M = 0.83, SD = 0.25), consistent with the demographic profile of allopathic medical students in the United States, who tend to come from relatively advantaged developmental backgrounds. The restricted range of PCE scores may have attenuated the observed associations. Future research should consider administering the BCEs-Revised (8) to obtain greater distributional sensitivity.

Seventh, year of training was included as a covariate and showed a marginal non-significant trend toward higher burnout in more advanced students (B = 0.130, *p* = 0.065). Although including year of training as a covariate partially addresses concerns about its confounding influence, a more complete treatment would involve testing whether year moderates the mediation model — specifically whether the indirect pathway from PCEs through belonging to burnout differs across M1-M4 students. Given sample size constraints, formal moderation by training year was not feasible and is recommended for future research with larger samples stratified across training phases.

### Future directions

The present findings open several important avenues for future research. First, longitudinal studies should examine the developmental trajectory of PCE-belonging-burnout associations across the full span of medical education, from matriculation through residency. Given that burnout tends to intensify in clinical training years (3), it is important to determine whether the protective associations of PCEs and belonging are stable, cumulative, or diminishing across training phases, and whether targeted belonging interventions during the transition to clinical clerkships can attenuate burnout escalation.

Second, future studies should test more complex multi-mediator models that simultaneously estimate the contributions of belonging, adaptive emotion regulation, self-compassion, and psychological flexibility as parallel or serial pathways through which PCEs are associated with burnout outcomes. This would allow researchers to compare the relative importance of interpersonal versus intrapersonal mechanisms and identify the most potent point of intervention.

Third, the equity implications of the PCE-belonging-burnout model warrant dedicated examination. Research should investigate whether sense of belonging functions as a stronger compensatory mediator for students with lower PCEs — a moderated mediation hypothesis that would identify the specific subpopulation for whom belonging-enhancing interventions are most critical.

Fourth, intervention research is urgently needed to test whether institutional belonging can be effectively cultivated through scalable pedagogical and cultural interventions, and whether such interventions reduce burnout in medical students. Randomized controlled designs evaluating peer mentorship programs, cohort learning communities, mindfulness-based group interventions, and faculty-student relational initiatives would provide the causal evidence necessary to translate the present correlational findings into evidence-based wellness policy.

Fifth, the marginal association between year of training and burnout observed in the present study warrants examination in larger samples with sufficient power to test training year as a moderator of the mediation model. Future studies should specifically examine whether the belonging-burnout pathway is stronger during clinical training years, and whether institutional belonging interventions at the M2-M3 transition — a period associated with heightened vulnerability — can attenuate normative burnout escalation.

## Conclusion

The present study provides initial cross-sectional evidence consistent with the hypothesis that sense of belonging may partially explain the statistical association between positive childhood experiences and burnout in medical students. The findings suggest — but do not demonstrate — that the developmental legacy of early nurturing environments may partly express itself in adult professional settings through students’ sense of institutional connection and belonging. These cross-sectional associations are theoretically coherent with developmental and occupational health frameworks, and they carry practical implications: since belonging, unlike PCEs, is institutionally modifiable, deliberate investment in inclusive, connected, and psychologically safe learning environments represents an evidence-informed — though not yet causally established — strategy for supporting medical student well-being. The marginal trend toward higher burnout in more advanced students further underscores that burnout prevention strategies must be responsive to the specific demands and vulnerabilities of each training phase, and that fostering belonging during the critical preclinical-to-clinical transition may be particularly valuable for sustaining the well-being of the next generation of physicians.

## Data Availability

The raw data supporting the conclusions of this article will be made available by the authors, without undue reservation.
